# Regional anesthesia might reduce recurrence and metastasis rates in adult patients with cancers after surgery: a meta-analysis

**DOI:** 10.1186/s12871-023-02400-w

**Published:** 2024-01-10

**Authors:** Shuang Xie, Liang Li, Fanqing Meng, Huanliang Wang

**Affiliations:** 1 Department of Anesthesiology, the Second Affiliated Hospital of Hainan Medical University, Haikou, Hainan China; 2 Department of Anesthesiology, Qilu Hospital of Shandong University, Jinan, Shandong China; 3 Department of Anesthesiology, Jinan Maternity and Child Health Care Hospital, Shandong First medical university, Jinan, China

**Keywords:** Regional anesthesia, General anesthesia, Cancer recurrence, Metastasis, Meta-analysis

## Abstract

**Background:**

The influence of anesthesia techniques on cancer recurrence and metastasis following oncological surgery is a topic of growing interest. This meta-analysis investigates the potential effects of regional anesthesia (RA), either independently or combined with general anesthesia (GA), on these outcomes.

**Methods:**

We performed an extensive search across PubMed, Embase, and the Cochrane Library databases. The primary outcome was cancer recurrence, while the secondary outcomes were local recurrence and distant metastasis. Pooled odds ratios (ORs) with 95% confidence intervals (CIs) were calculated by utilizing random-effects models. The Newcastle-Ottawa Scale (NOS) was used for quality assessment of observational studies, the Cochrane Risk of Bias Tool for Randomized Trials (Rob 2.0) was used for randomized controlled trials, and all the outcomes were assessed by using the Grading of Recommendations, Assessment, Development and Evaluation (GRADE).

**Results:**

This study included 32 studies comprising 24,724 cancer patients. RA, either alone or in combination with GA, was significantly associated with reduced cancer recurrence compared to GA alone (OR = 0.82; 95% CI = 0.72 to 0.94; *p* < 0.01). This association remained significant for prostate cancer patients in subgroup analyses (OR = 0.71; 95% CI = 0.51 to 0.98; *p* = 0.04) and in the context of epidural anesthesia combined with GA. However, there were no significant associations noted for local recurrence or distant metastasis.

**Conclusions:**

This meta-analysis provides evidence that RA, used alone or adjunctively with GA, is associated with a lower risk of cancer recurrence, particularly in patients with prostate cancer. However, no significant effects were observed on local recurrence or distant metastasis. Further prospective studies should be conducted to clarify this important issue.

**Supplementary Information:**

The online version contains supplementary material available at 10.1186/s12871-023-02400-w.

## Background

Cancer ranks as a leading cause of mortality for diseases worldwide, with 10 million cancer deaths worldwide in 2020 [1]. Surgical resection is a mainstay therapy for cancer. Surgery can’t be conducted without anesthesia, but different anesthesia techniques could affect the recurrence and metastasis of cancer after surgery [2–4]. For example, the addition of regional anesthesia (RA) to general anesthesia (GA) is proven to be beneficial to postoperative oncological outcomes compared with GA alone among patients with prostate cancer [2] or breast cancer [3]. Besides, RA alone (spinal anesthesia) was associated with a lower 5-year tumor recurrence rate compared with GA after transurethral resection of bladder tumors [5].

RA includes spinal anesthesia, epidural anesthesia, local anesthesia infiltration, and nerve block. RA can largely attenuate the neuroendocrine stress response to surgery by reducing the catecholamine levels and minimizing immunosuppression [6], which can not only provide effective pain control but also reduce exposure to opioids; in return, it reduces the potential effects of the latter on postoperative prognosis [7, 8]. Furthermore, similar findings have been shown in animal models [9, 10].

However, studies on the impact of RA on cancer recurrence and metastasis yielded negative and positive results. For example, some studies have reported that RA with or without GA was not significantly associated with a lower incidence of cancer recurrence and metastasis rate than GA in cancer resection surgery [11–14]. Some meta-analyses [15–17] investigated the impact of RA with or without GA on cancer recurrence and metastasis, and the results indicated that RA with or without GA did not reduce cancer recurrence and metastasis rate after surgery. These results of meta-analysis should be interpreted with caution due to the low level of evidence, such as a limited number of studies (N ≤ 10), no adjusted for different types of cancer (breast cancer, colorectal cancer, and prostate cancer), and cancer recurrence (local recurrence and distant metastasis).

Given existing individual studies [2–4], and contrasting evidence from previous meta-analyses [15–17], the present study aimed to conduct a comprehensive meta-analysis to investigate the impact of RA on the incidence of cancer recurrence and metastasis rate after surgery. To provide more detailed insights, we also conducted subgroup analyses based on cancer types [2–4]and cancer recurrence types [15–17]. Based on results presented in existing literature, we hypothesize that regional anesthesia (RA) may have an impact on cancer recurrence and metastasis rate after surgery, and this impact may vary depending on the type of cancer or the type of cancer recurrence.

## Methods

The meta-analysis was performed according to the Preferred Reporting Item for Systematic Reviews and Meta-analysis (PRIMA) [18]. This study is registered with the PROSPERO registry, number CRD42022370267.

### Search strategy

Literature was retrieved through PubMed, Embase, and the Cochrane Library (updated to August 29, 2022) using the following keywords: neoplasms, cancer, tumor, local anesthesia, regional anesthesia, epidural anesthesia, recurrence, metastasis, prognosis, and survival. Besides, we searched the reference list of relevant reviews and eligible studies to identify additional studies.

### Inclusion and exclusion criteria

Inclusion and exclusion criteria in the present study were based on the Population, Intervention, Comparator, Outcomes, and Study designs (PICOS) structure.

1. Population: patients who underwent any type of cancer resection surgery. Adults only.
2. Intervention: a comparison of the use of regional anesthesia, regardless of types of RA.
3. Comparator: versus general anesthesia, regardless of volatile anesthesia or total intravenous anesthetic agents.
4. Outcome: studies reported rates of cancer recurrence or metastasis after surgery.
5. Study design: any prospective or retrospective cohort, case-control observational studies, and randomized controlled trials (RCTs).

Besides, Reviews, meta-analyses, conference abstracts, animal trials, and studies that did not provide sufficient data were excluded.

### Data extraction

Two independent reviewers extracted the essential data. We extracted the following data from each eligible study: the first author, publication year, study design, cancer type, sample size and the number of patients assigned in each group, RA techniques, median follow-up time, whether propensity score matching or not and the information of methodological quality. Whenever discrepancies in data extraction occurred, the consensus was achieved through discussion or consulting a third reviewer.

### Outcomes

The primary outcome was defined as post-operative cancer recurrence or metastasis rate as reported by the study authors. Cancer recurrence is defined as the emergence of a new tumor at or near the original tumor site after treatment. Depending on the location, it is classified into local recurrence and distant metastasis. Local recurrence refers to the reappearance of cancer at or near the original site. Distant metastasis refers to the spread of cancer cells from the original site to other parts of the body. The secondary outcomes included subgroup analyses based on cancer types, cancer recurrence types, anesthetic technique, and study design [19].

### Quality assessment

Quality assessment of the included studies will be carried out independently by two reviewers and any disagreements will be resolved through discussion or consultation with a third reviewer.

The Newcastle-Ottawa Scale (NOS) was used to assess the methodological quality of the observational studies [20]. NOS contains three dimensions, including patient selection (three items), comparability of the two study arms, and assessment of the outcomes (two items). The total points ranged from 0 to 9 stars. Generally, 0–4 points were considered poor quality, 5–6 points as moderate quality, and 7–9 points as high quality.

The Cochrane Collaboration’s tool for assessing the risk of bias (ROB-2) [21]. The following five dimensions are included: bias arising from the randomization process, bias due to deviation from the intended intervention, bias due to missing outcome data, bias in the measurement of the outcome, and bias in the selection of reported results. Each of these aspects will be labeled as high risk, some concern, and low risk, depending on the degree of match between the facts presented in the eligible studies and the assessment criteria. The overall level will be labeled as low risk, some concern, or high risk, depending on the results of the assessment in each of the five categories. Any disagreement between the two authors on the risk of bias assessment will be resolved through discussion to reach an agreement.

The certainty of evidence for each study was graded according to the Grading of Recommendations Assessment, Development, and Evaluation (GRADE) working group method [22]. This method considers the study design, risk of bias, inconsistency, indirectness, imprecision, and other factors to grade the level of certainty as high, moderate, low, or very low.

### Statistical analysis

All analyses were performed using the STATA SE 14.0 software (StataCorp, College Station, Texas, USA). The odds ratio (OR) and corresponding 95% confidence interval (CI) were used to summarize the results. The Q-test and I^2^ statistic were used to describe heterogeneity among studies. If the I^2^ value was over 50%, indicating significant heterogeneity, a random-effects model was used. Conversely, a fixed-effects model was utilized when the I^2^ value was 50% or less. I2 values of > 75%, 25-75% and < 25% were defined as high, moderate, and low heterogeneity, respectively. Subgroup analysis was used to explore possible sources of heterogeneity. Sensitivity analysis by leave-one-out method was used to test the robustness of the results. Publication bias was assessed using funnel plots and Egger’s test, and if significant bias was present, trim-and-fill analysis was used to account for any potential missing studies. *P* < 0.05 indicated statistical significance.

## Results

### Study selection

**Fig. 1:**
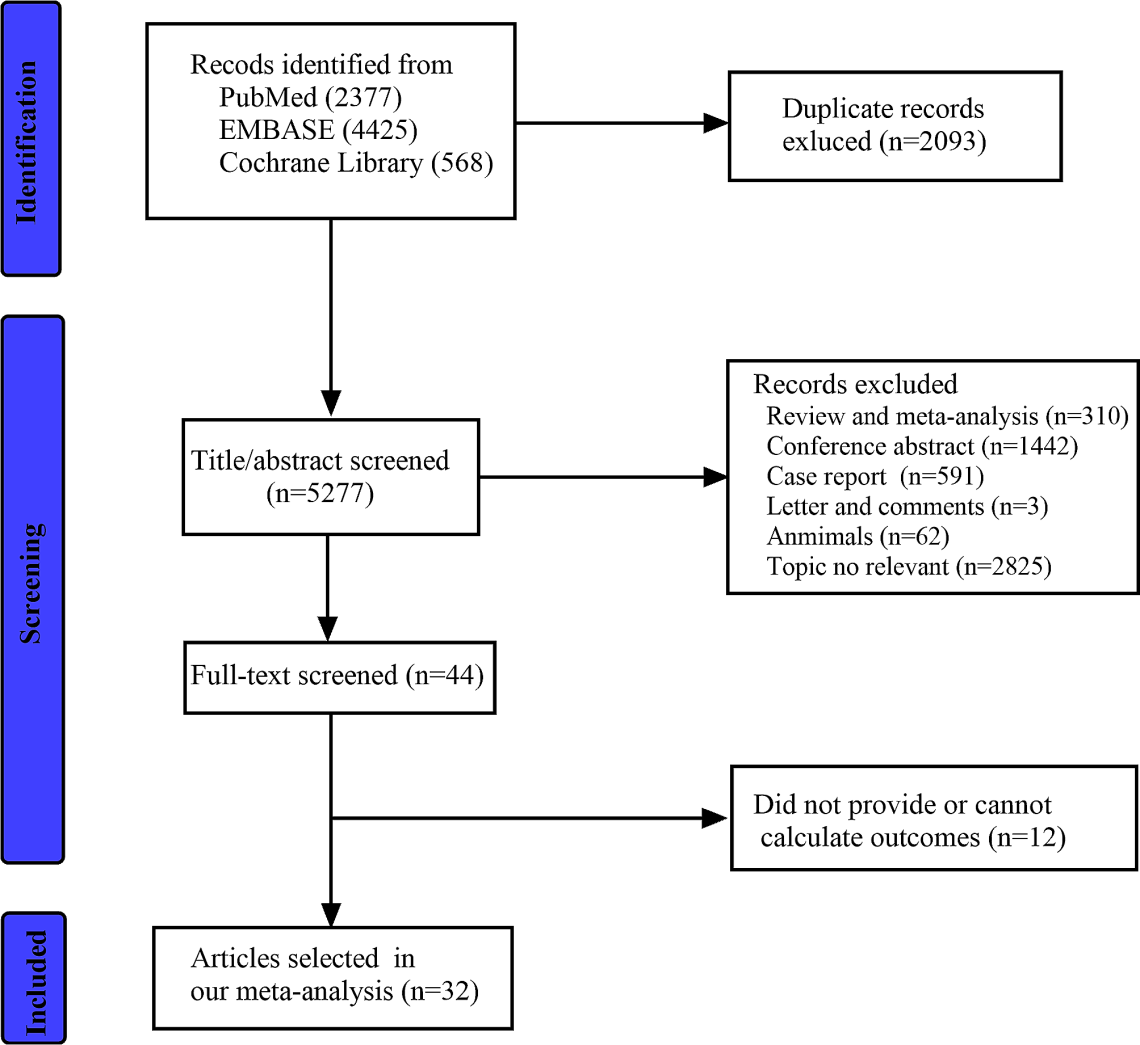
PRISMA flow chart for study screening and inclusion

A total of 7370 studies were retrieved as potentially relevant literature reports through the initial searches in PubMed, Embase, and the Cochrane Library databases, and 2093 duplicate studies were deleted. Then, 5233 kinds of literature were excluded after reviewing the title or abstract. After retrieving 44 full-length articles, ultimately, 27 studies [2, 3, 5, 11–14, 23–42] were eligible for data extraction and meta-analysis. Besides, five studies [4, 43–46] were in our meta-analysis by manual search. The study selection process is presented in Fig. 1.

### Study characteristics

The characteristics of the eight included studies are summarized in Table 1. In our study, 32 articles were included, involving 24,724 cancer patients. The sample size of each included study ranged from 91 to 5960. Of all the included studies, 24 were retrospective cohort studies, five were RCTs, two were prospective cohort studies, and one was a cross-sectional study. Cancer types included bladder cancer, breast cancer, colorectal cancer, esophageal cancer, gastric cancer, hepatocellular carcinoma, ovarian cancer, and prostate cancer. A total of 12 studies investigated the association between only RA and GA on cancer recurrence and metastasis rate, and 20 studies examined the association between RA + GA and GA on cancer recurrence and metastasis rate.

**Table 1:** Characteristics of included studies

Study	Study design	Cancer type	Age,mean (Intervention , Control)	Sample size (I ,C)	RA technique	PSM	Certainty of the evidence (GRADE)	length of follow up(month)
Gupta 2011	Retrospective cohort	Colorectal cancer	71.4,73.2	655(562,93)	EA + GA	No	Low	31
Wuethrich 2010	Retrospective cohort	Prostate cancer	63,64	261	EA + GA	No	Low	102
Li 2022	RCT	Breast cancer	48 ± 10 49 ± 9	1253	PVB + GA	No	High	53
de Oliveira 2011	Retrospective cohort	Ovarian cancer	55,57	183	EA + GA	No	Low	42 (IQR12-60)
Mu 2021	Retrospective cohort	Colorectal cancer	60.5 ± 10.5, 61.2 ± 12.8	174	EA + GA	Yes	Low	41(IQR39-43)
Tsui 2010	RCT	Prostate cancer	63.0 ± 5.5 63.9 ± 6.1	99	EA + GA	No	High	54
Wuethrich 2013	Retrospective cohort	Prostate cancer	63.8,63.6	148	EA + GA	No	Low	135 (IQR 14–198)
Hasselager 2022	Prospective cohort	Colorectal cancer	70,70	5960	EA + GA	Yes	Moderate	NA
Macleod 2018	Prospective cohort	Prostate cancer	59.5,60	2909	PVB + GA	No	Moderate	No multimodal analgesia:135 (IQR109–150) Multimodal analgesia: 55 (IQR29–83)
Biki 2008	Retrospective cohort	Prostate cancer	63 ± 5, 62 ± 6	225	EA + GA	No	Low	33–153
Karmakar 2017	RCT	Breast cancer	52,51	177	PVB + GA	No	High	60
Sessler 2019	RCT	Breast cancer	53,53	2108	EA/LA/SA + GA	No	High	36 (IQR 24–49)
Christopherson 2008	RCT	Colorectal cancer	68.6 ± 7.7, 69.1 ± 7.8	177	EA + GA	No	High	NA
Pei 2020	Retrospective cohort	Gastric cancer	65,75	194	EA + GA	Yes	Low	NA
Exadaktylos 2006	Retrospective cohort	Breast cancer	NA	129	PVB + GA	No	Low	32 ± 5
Gottschalk 2010	Retrospective cohort	Colorectal cancer	65,63	509	EA + GA	No	Low	21(IQR9-46)
Kuo 2014	Retrospective cohort	Hepatocellular carcinoma	63.7 ± 10.7, 64.7 ± 11.7	118	SA	No	Low	24
Lai et al. 2012	Retrospective cohort	Hepatocellular carcinoma	51.5 ± 16.6, 54.9 ± 11.3	179	EA	No	Low	43(IQR2-129)
Koumpan 2018	Retrospective cohort	Bladder Cancer	71.7 ± 10.5, 65.4 ± 10.5	243	SA	No	Low	NA
Tseng 2014	Retrospective cohort	Prostate cancer	58,58	1964	SA	No	Low	48 to 60
Heinrich 2015	Retrospective cohort	Esophageal cancer	61,61	153	EA + GA	No	Low	NA
Hiller 2014	Retrospective cohort	Gastro-oesophageal cancer	67,66	140	EA + GA	No	Moderate	NA
Holler 2013	Retrospective cohort	Colorectal cancer	NA	749	EA + GA	No	Low	NA
Zhang 2021	Retrospective cohort	Breast cancer	54.1, 54.1	2790	PVB-RA	Yes	Low	patients receiving INHA-GA without propofol: 61.2 ± 25.2 patientsreceiving PB-RA with propofol: 62.1 ± 28.1
Sprung 2014	Retrospective cohort	Prostate cancer	63.9, 63.9	387	EA	No	Low	NA
Capmas 2012	Retrospective cohort	Ovarian cancer	56, 56	94	EA + GA	No	Low	33 to 153
Wang 2020	Retrospective cohort	Hepatocellular carcinoma	57.6, 56.1	489	EA/LA/SA	No	Low	NA
Choi 2017	Retrospective cohort	Bladder Cancer	63 ± 12, 61 ± 13	690	SA	Yes	Low	35 (IQR 11–57)
Lu 2021	Retrospective cohort	Breast cancer	45, 45	169	EA	No	Low	more than 60
Karanlik 2017	Case-control	Breast cancer	72.4 ± 6, 71.1 ± 3.7	91	LA	No	Low	GA :55.09 ± 13.49 (IQR 38–104) LA: 58.7 ± 15.5 (IQR 20–99)
Lin 2011	Retrospective cohort	Ovarian cancer	45.7, 48.1	143	EA	No	Low	24 to 174
Lee 2022	Retrospective cohort	Bladder Cancer	66.8 ± 6.1, 66.5 ± 6.1	1164	EA/LA/SA	Yes	High	53 ± 21

EA, epidural anesthesia; GA, general anesthesia; LA, local anesthesia; NOS, Newcastle-Ottawa Scale; PSM, propensity score matching; PVB, paravertebral block; RA, regional anesthesia; RCT, randomized control trial; SA, spinal anesthesia. NA: Not Available. IQR: Inter-Quartile Range

GRADE Working Group grades of evidence

High certainty: we are very confident that the true effect lies close to that of the estimate of the effect

Moderate certainty: we are moderately confident in the effect estimate: the true effect is likely to be close to the estimate of the effect, but there is a possibility that it is substantially different

Low certainty: our confidence in the effect estimate is limited: the true effect may be substantially different from the estimate of the effect

Very low certainty: we have very little confidence in the effect estimate: the true effect is likely to be substantially different from the estimate of effect

### Quality assessment

Observational studies were assessed using the Newcastle-Ottawa Scale, and all included studies were of acceptable quality.20 studies were considered to be of high quality and 7 were considered to be of moderate quality (Supplementary Table 2). For randomized controlled studies, methodological quality was assessed using the Cochrane Collaboration’s Risk of Bias Assessment Tool (ROB 2.0). All RCTs were considered to be at low risk except one [23] (Supplementary Table 3). Each study was assessed using GRADE and the results are shown in Table 1.

### Cancer recurrence

**Fig. 2:**
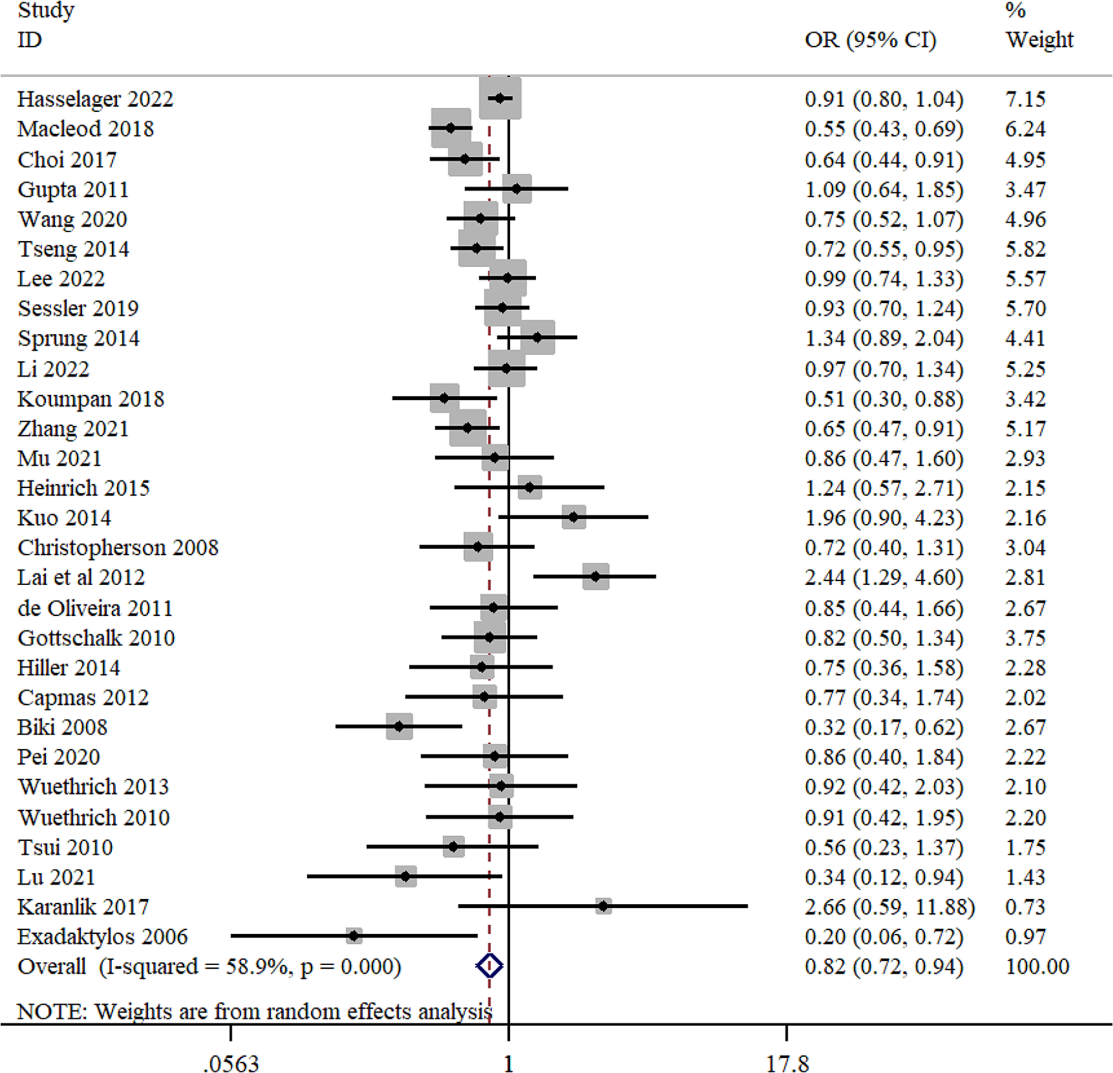
Forest plot for cancer recurrence

Twenty-nine studies provided suitable data for cancer recurrence. The pooled OR of cancer recurrence showed a significant difference between RA with or without GA and GA groups (OR = 0.82; 95%CI = 0.72 to 0.94; *I*^2^ = 58.9%) (Fig. 2).

Subgroup analysis reported that significant associations were also observed in prostate cancer (OR = 0.71; 95%CI = 0.51 to 0.98; *I*^2^ = 70.2%) (Table 2). Furthermore, significant associations were also observed in subgroup analysis based on anesthesia technique (epidural anesthesia with GA: OR = 0.87; 95%CI = 0.79 to 0.97; *I*^2^ = 0.0%) (Table 2), and study design (retrospective cohort: OR = 0.82; 95%CI = 0.69 to 0.98; *I*^2^ = 56.8%) (Table 2). Also, all the subgroup differences were not statistically significant (*P* > 0.05) (Table 2).

**Table 2:** Subgroup Analyses for cancer recurrence and metastasis

Subgroup	Number	Pooled OR (95%CI)	*P*-value for heterogeneity and *I*^2^	*P*-value for subgroup difference
Recurrence				
Cancer type				0.977
Bladder Cancer	3	0.72 (0.49, 1.05)	0.05 and 66.7%	
Breast cancer	6	0.75 (0.53, 1.07)	0.019 and 62.9%	
Colorectal cancer	5	0.91 (0.81, 1.02)	0.876 and 0.0%	
Gastric/esophageal cancer	3	0.92 (0.60, 1.43)	0.641 and 0.0%	
Hepatocellular carcinoma	3	1.47 (0.64, 3.38)	0.002 and 84.2%	
Ovarian cancer	2	0.82 (0.49, 1.37)	0.853 and 0.0%	
Prostate cancer	7	0.71 (0.51, 0.98)	0.003 and 70.2%	
Anesthesia technique				0.428
PVB + GA	3	0.60 (0.34, 1.06)	0.004 and 81.9%	
SA	4	0.75 (0.52, 1.08)	0.039 and 64.2%	
EA	3	1.14 (0.48, 2.71)	0.005 and 80.9%	
EA + GA	14	0.87 (0.79, 0.97)	0.495 and 0.0%	
Study design				0.653
Prospective cohort	2	0.71 (0.43, 1.18)	< 0.001 and 93.1%	
RCTs	4	0.90 (0.74, 1.09)	0.607 and 0.0%	
Retrospective cohort	22	0.82 (0.69, 0.98)	0.001 and 56.8%	
Local recurrence				
Cancer type				-
Breast cancer	4	0.49 (0.19, 1.26)	0.199 and 35.6%	
Anesthesia technique				-
EA + GA	2	0.94 (0.46, 1.91)	0.519 and 0.0%	
Study design				-
Retrospective cohort	6	0.78 (0.43, 1.45)	0.053 and 54.3%	
Distant metastasis				
Cancer type				0.265
Breast cancer	6	0.73 (0.47, 1.11)	0.317 and 15.2%	
Colorectal cancer	3	0.96 (0.72, 1.28)	0.311 and 0.0%	
Anesthesia technique				0.754
PVB + GA	2	0.67 (0.27, 1.70)	0.361 and 0.0%	
EA	2	0.37 (0.01, 19.94)	0.006 and 86.5%	
EA + GA	5	0.94 (0.73, 1.20)	0.653 and 0.0%	
Study design				0.480
RCTs	8	0.90 (0.69, 1.18)	0.145 and 35.5%	
Retrospective cohort	3	0.69 (0.39, 1.22)	0.822 and 0.0%	

EA, epidural anesthesia; GA, general anesthesia; PVB, paravertebral block; RCTs, randomized control trials; SA, spinal anesthesia

### Local recurrence

**Fig. 3:**
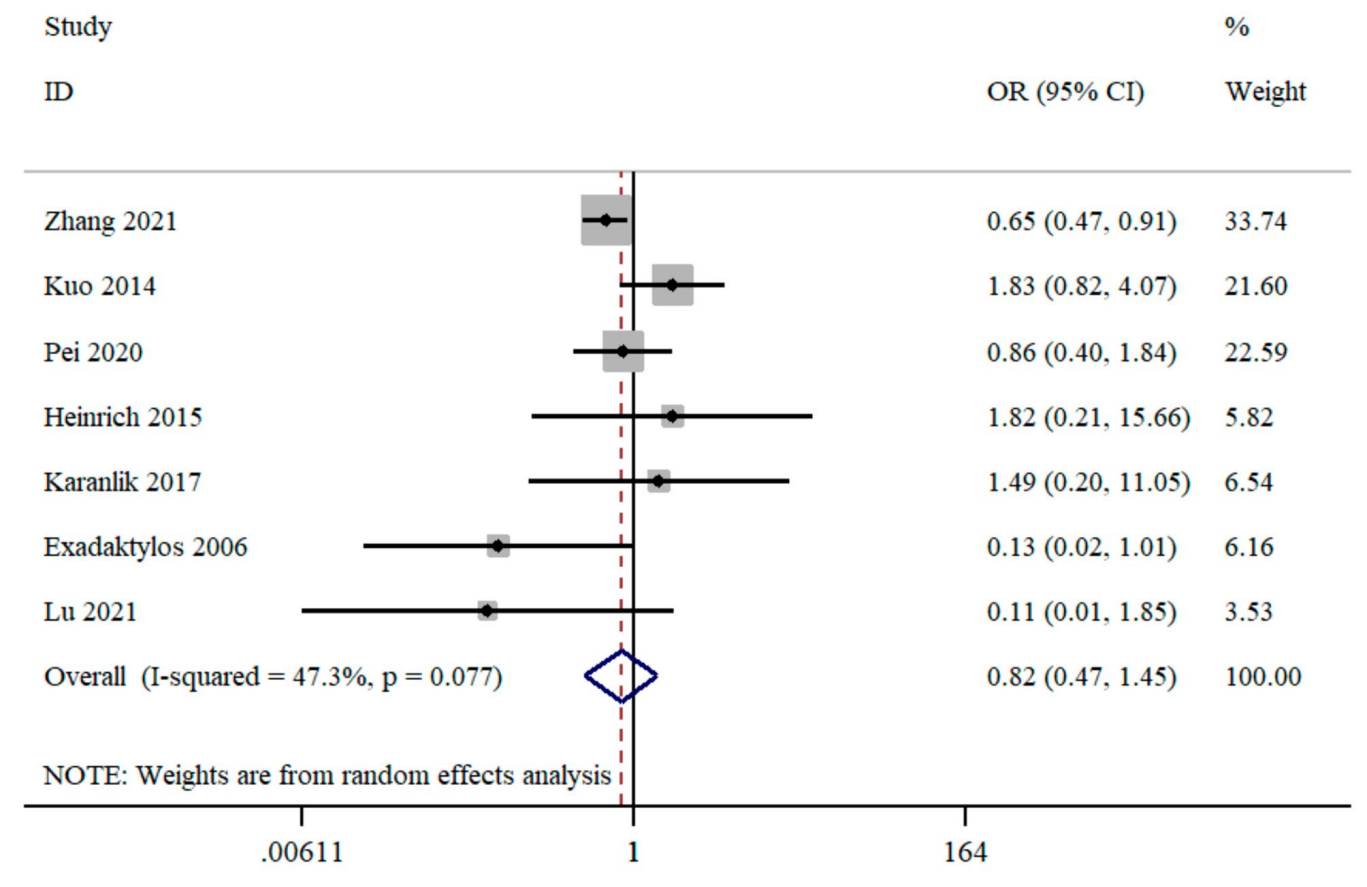
Forest plot for cancer local recurrence

A total of seven studies provided suitable data for local recurrence. No significant positive association between RA with or without GA and GA groups in local recurrence (OR = 0.82; 95%CI = 0.47 to 1.45; *I*^2^ = 47.3%) (Fig. 3). Subgroup analysis reported that no significant associations were also observed in breast cancer, epidural anesthesia with GA, and retrospective cohort studies (Table 2).

### Distant metastasis

**Fig. 4:**
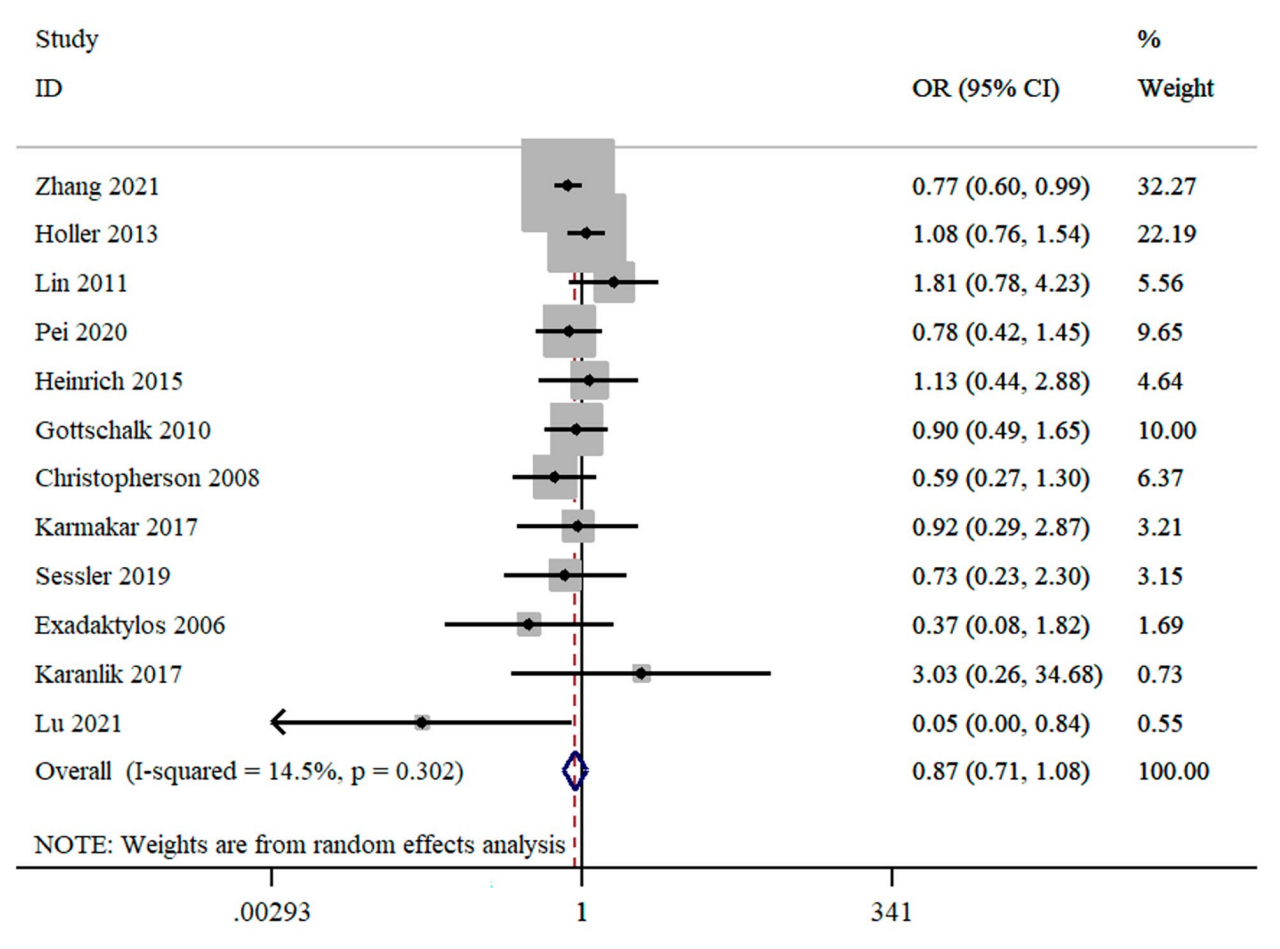
Forest plot for cancer metastasis

Twelve studies provided suitable data for distant metastasis. The pooled OR of distant metastasis showed no significant difference between RA with or without GA and GA groups (OR = 0.87; 95%CI = 0.71 to 1.08; *I*^2^ = 14.5%) (Fig. 4). Subgroup analysis reported that no significant associations were also observed based on cancer type, anesthesia technique, and study design (Table 2). Again, all the subgroup differences were not statistically significant (*P* > 0.05) (Table 2).

### Publication bias and sensitivity analysis

Sensitivity analyses showed that the pooled effect size results were robust (Supplementary Figs. 4–6). Funnel plot and Egger’s test were used to evaluate the publication bias of the included studies in the meta-analysis. The funnel plot did not reveal any evidence of asymmetry (Supplementary Figs. 1–3). Egger’s tests were not significant, indicating the absence of publication bias among the included studies (Table 3).

**Table 3:** Egger’s test for publication bias

Outcomes	Number	Egger’s test
Recurrence	29	0.831
Local recurrence	7	0.978
Distant metastasis	12	0.768

## Discussion

Our meta-analysis, which included a comprehensive collection of 32 studies, revealed a significant difference in cancer recurrence between groups receiving RA with or without GA and groups receiving GA alone. Specifically, the pooled OR for cancer recurrence was found to be 0.82 (95% confidence interval (CI) = 0.72 to 0.94), suggesting a lower risk of cancer recurrence in patients receiving RA alone or concurrent GA. Furthermore, subgroup analyses underscored the significance of this finding in prostate cancer, epidural anesthesia with GA, and retrospective cohort studies.

A growing number of studies have found that anesthesia techniques could affect the recurrence and metastasis of cancer after surgery [2–4]. However, a previous meta-analysis [16, 17] suggested that RA with or without GA did not reduce cancer recurrence and metastasis, which was inconsistent with individuals’ studies [2–4]. Therefore, our study conducted a large-scale meta-analysis to investigate the impact of RA on postoperative cancer recurrence and metastasis.

The present meta-analysis indicated that compared with GA, the use of RA alone or in combination with GA was significantly associated with cancer recurrence, but specifically, no significant association was found in cancer metastasis and local recurrence. Anesthetics are commonly used in the operative treatment of tumors. The choice of different anesthetics and anesthesia techniques can affect cancer proliferation, metastasis, recurrence, and prognosis. It is hypothesized that one of the mechanisms by which RA reduces cancer recurrence is through anti-inflammatory effects and reduction of surgical stress response [47]. For example, some studies found a small-modest reduction in inflammatory biomarkers (i.e., interleukin 1 [IL-1], IL-6, MMP-3, and MMP-9) and markers of the stress response (i.e., serum cortisol, serum glucose, and C-reactive protein) in patients who received a paravertebral block (PVB) [48–50], which supported the hypothesis. Studies found that RA can not only reduce the number of opioids [51] but also inhibit tumor recurrence by blocking sodium channels of cancer cells [52], decreasing inflammation [53], and improving immune function [54]. Studies suggested that opioids can be beneficial to tumor growth by inducing immune suppression and stimulating the proliferation of cancer metastasis [51]. Therefore, the American Society of Anesthesiologists (ASA) advocated minimizing the use of opioids in cancer patients.

A second mechanism by which regional anesthesia reduces postoperative cancer recurrence is decreasing the concentration of growth factors with proliferative or angiogenic effects. For example, Jaura et al. [55] and Deegan et al. [56] found that serum from breast cancer women treated with sevoflurane/opioids was antiapoptotic, whereas serum from women treated with PVB/propofol drugs was inhibitory to cell proliferation. Besides, it has been hypothesized that RA attenuates the inhibitory effects of surgery itself, volatile anesthetics, and opioids on these cells. Furthermore, inhaled anesthetics and intravenous opioids may inhibit the activity of natural killer (NK) and functional T cells for several days [57–59]. However, RA can maintain NK cell function in tumor patients [60]. However, prior some meta-analyses have indicated that RA with or without GA did not reduce cancer recurrence and metastasis rate after surgery [16, 17], which did not follow our results. These mate analyses are based on a few original studies (N ≤ 10), which may cause unstable results. For example, Lee et al. [17] only recruited three studies to calculate the pooled OR of cancer recurrence between RA and GA. Ang et al. [17] also only included six studies in the meta-analysis.

Furthermore, within the subgroup of prostate cancer patients, RA with or without GA was revealed to be associated with lower cancer recurrence, but the same result was not found in a subgroup analysis of cancer type. The previous meta-analysis was in agreement with our findings. For example, Pei et al. [16] found that general-epidural anesthesia (EGA) might be associated with cancer-free survival benefits among patients with operable prostate cancer; however, no significant benefits were detected in colorectal cancer. Besides, Lee et al. demonstrated that the use of regional analgesia contributed to improving overall survival in patients after prostatectomy [61]. The incidence of postoperative cancer recurrence may depend on the nature and different types of cancer. Biochemical recurrence rates for prostate cancer range from 20 to 40%, which is significantly lower than more aggressive cancer types such as hepatocellular carcinoma (HCC) [62–64]. In the present study, our results also indicated that EA could decrease the cancer recurrence rate in cancer resection surgery, compared with GA, which was consistent with previous studies [16]. Animal models have reported that EA could improve perioperative immune suppression and enhance immune surveillance among cancer patients, thereby decreasing cancer recurrence [9]. We note that the larger RCTs related to breast cancer in the included studies did not show a difference. Zhang and Du raised a similar issue [11, 42, 65–70], that regional anesthesia has a beneficial effect on breast cancer recurrence compared with general anesthesia, but this effect has only been reported in some observational studies and research (in vitro), not in RCTs (including this review). The reasons for this may be the huge differences in the duration between anesthesia experiments (in vitro) and clinical application of anesthesia [71], as well as the biological characteristics of different cancers [72]. In addition, there were RCTs believed that regional anesthesia was effective for the recurrence of the surgery whose wound is large, while breast cancer surgery is less invasive [23, 73]. In addition, the weights of these five RCTs included in this study, Li (5.25%), Tsui (1.75%), Sessler (5.7%), Christo (3.04%), and Karmakar (3.21%), were not overwhelming, which may be one of the reasons why the larger RCTs included did not show differences. Finally, all sensitivity analyses showed that the pooled effect size results were robust.

Although the impact of RA on cancer recurrence was inconclusive, our study supported that the use of RA was associated with a lower incidence of cancer recurrence rate than GA in cancer resection surgery. However, our findings should be interpreted with caution due to some limitations. First, there were only five RCTs although 32 studies were included. Therefore, our meta-analysis was limited by the nature of the nonrandomized and retrospective studies with significant heterogeneity and low-quality evidence. Second, our study did not control some other confounding variables, such as changes in the definition of recurrence, and different lengths of follow-up, which hampers our conclusions. Third, 31 studies in the English language were included in the present meta-analysis, which introduces “English language bias” and reduces the accuracy of our results. Fourth, the title of this study was adjusted according to the results of the studies compared to the registration, and the original title was “Anesthesia type may impact on cancer recurrence and metastasis after cancer surgery: a meta-analysis”. In addition, the RCT quality assessment method was adjusted from NOS to ROB2.0. Given the limited and heterogeneous evidence, it may be too early to change the anesthesia practice in surgeries for cancer. However, we believe our findings provided a reference for future studies in this area.

Recently, the incidence of cancer has gradually increased, and although the mortality rate has decreased with the increasing maturity of treatment, the mortality rate is still at a high level, so inhibiting tumor recurrence and metastasis and increasing the survival rate of patients with tumors have become the focus of people’s research. Although we found a slight apparent advantage of regional anesthesia in some subgroups, these findings should be interpreted cautiously when formulating hypotheses because the combined effects in subgroups were derived from a small number of original studies and were not corrected for multiple comparisons. Given the study limitations and various findings, it may be too early to change anesthetic practices in cancer surgery. Still, we believe that our findings provide recommendations for future research in this field.

## Conclusions

In conclusion, our meta-analysis indicated that RA may be associated with lower cancer recurrence in cancer patients after surgery, especially for these prostate cancer patients. Furthermore, our results suggested a significant positive association between EGA and cancer recurrence. However, no significant findings were found in cancer metastasis and local recurrence. Further prospective studies should be conducted to clarify this important issue.

### Electronic supplementary material

Below is the link to the electronic supplementary material.

Supplementary Material 1

Supplementary Material 2

## Data Availability

All data generated or analysed during this study are included in this published article.
