# Supplementary material for: Regional anesthesia might reduce recurrence and metastasis rates in adult patients with cancers after surgery: a meta-analysis
Source: BMC Anesthesiol. 2024 Jan 10;24:19. doi: 10.1186/s12871-023-02400-w (PMC10777509; doi:10.1186/s12871-023-02400-w)
Supplement: Supplementary file 2 — Supplementary Material 2 [file 12871_2023_2400_MOESM2_ESM.pdf]

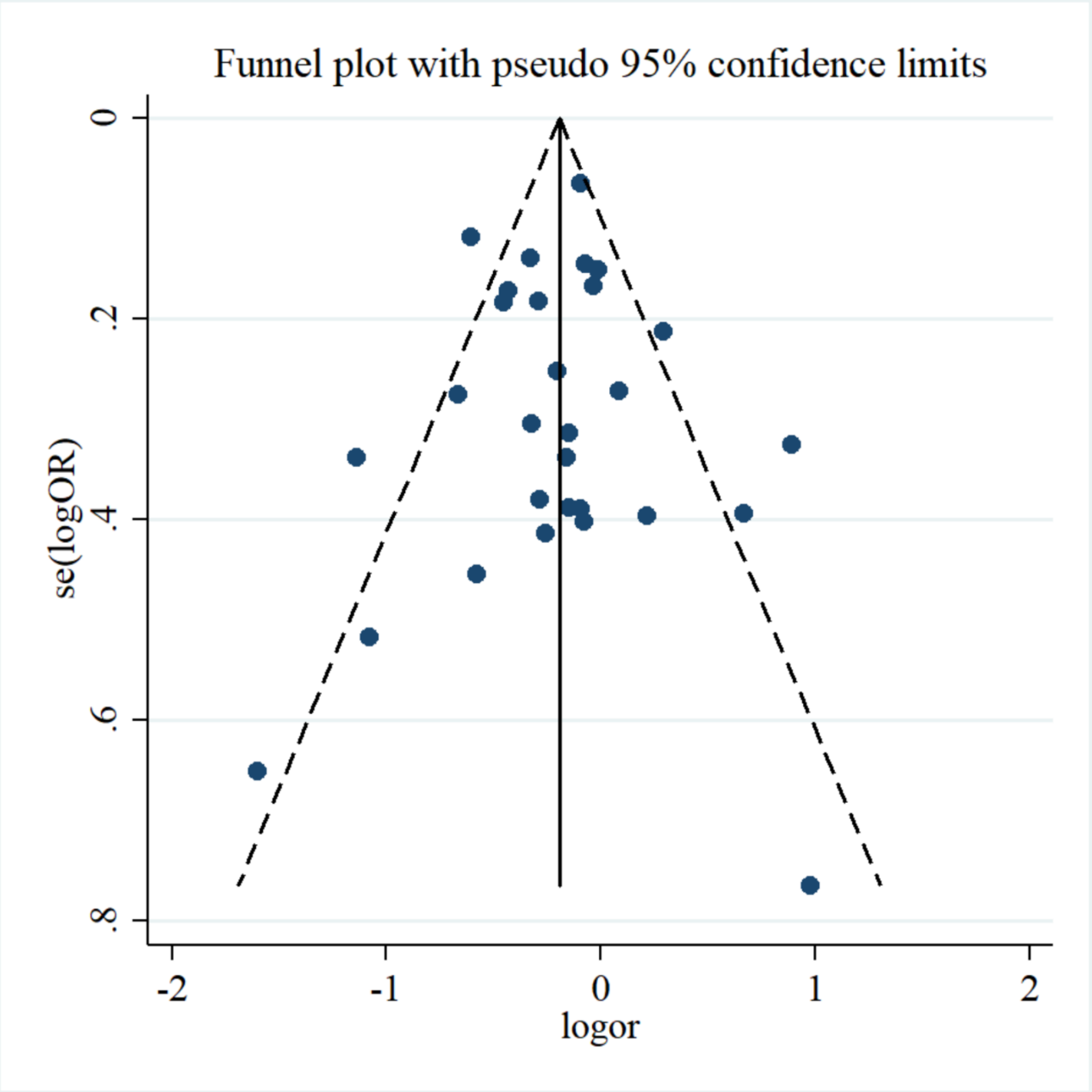

S Figure 1. Funnel plot for cancer recurrence.

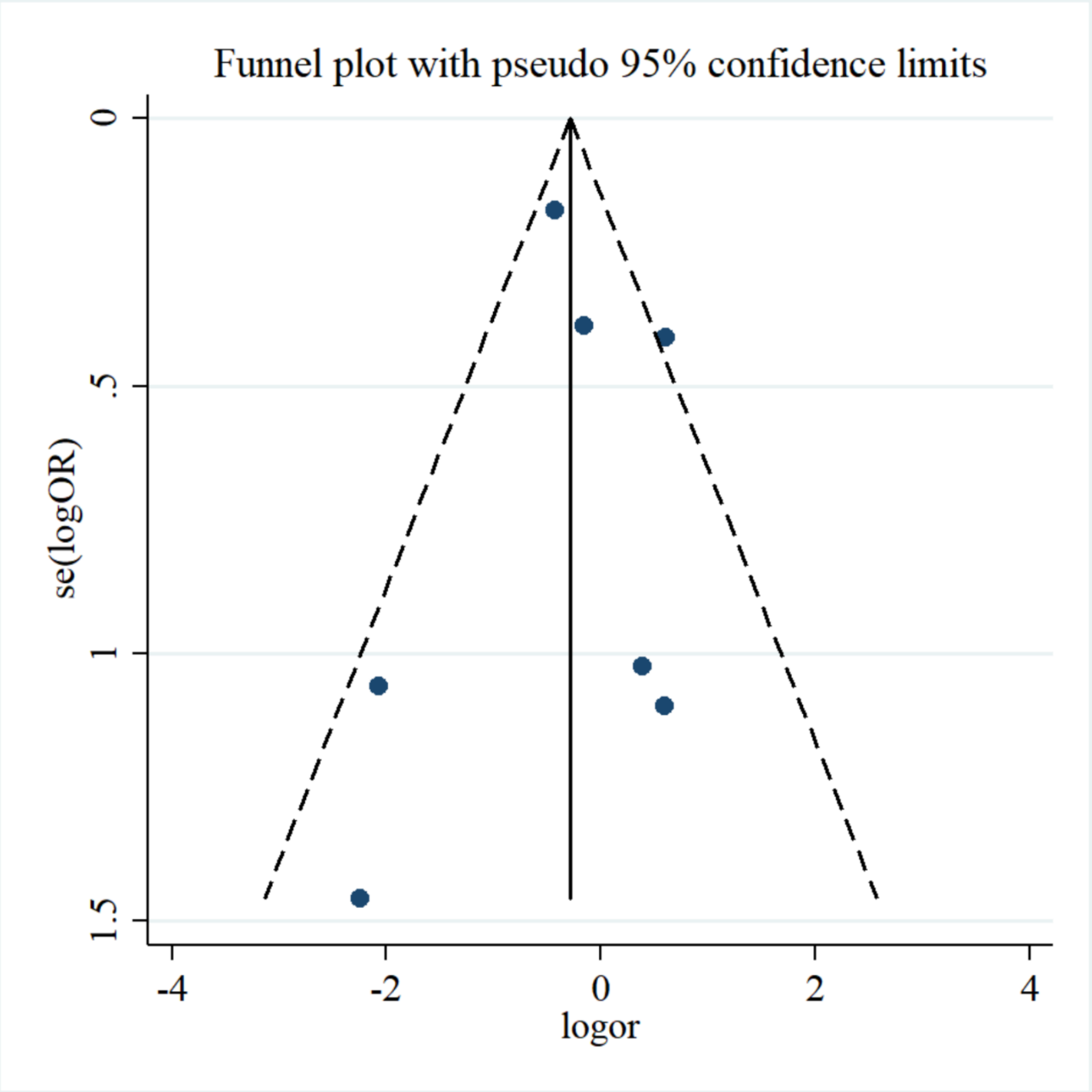

S Figure 2. Funnel plot for cancer local recurrence.

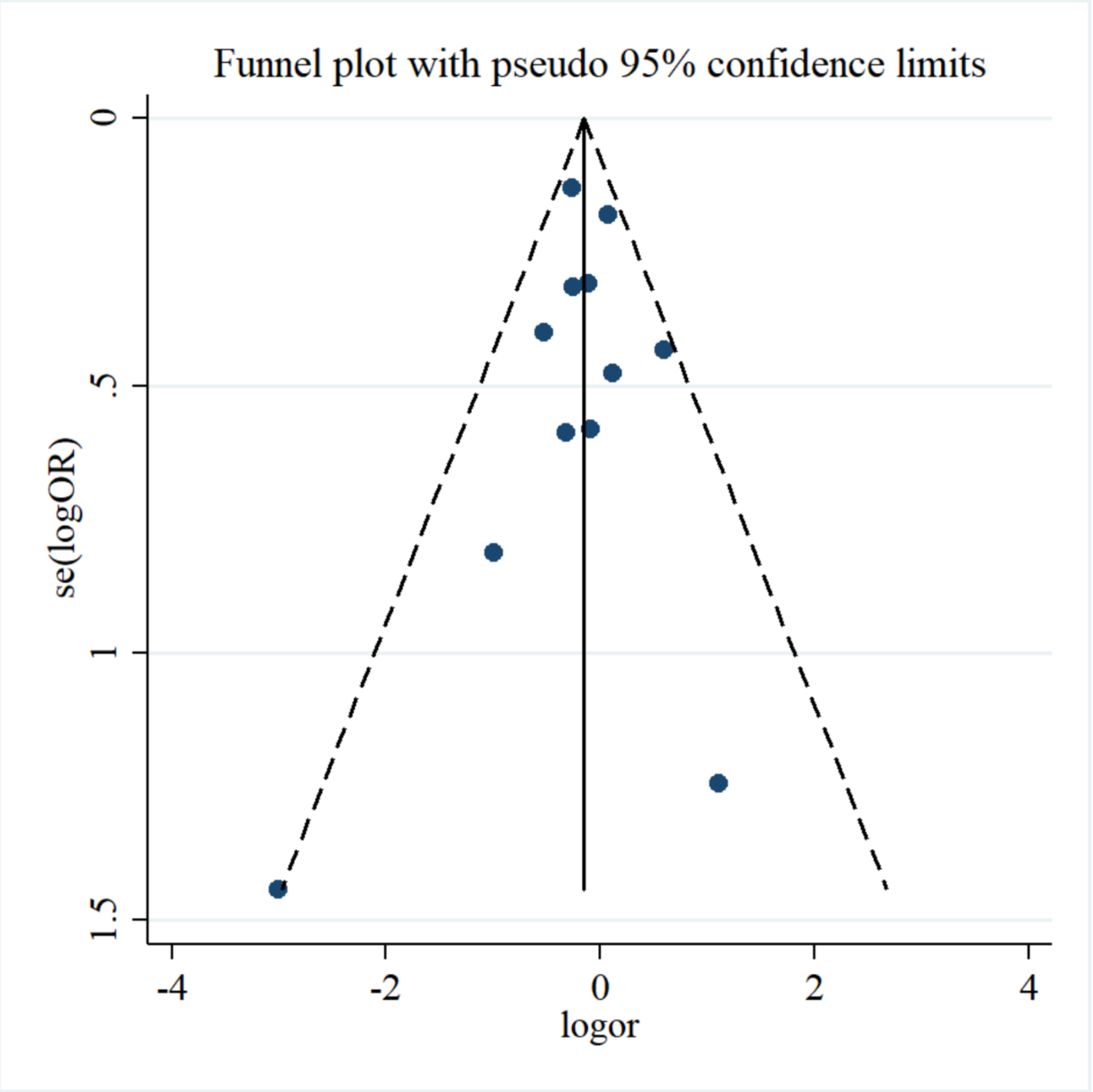

S Figure 3. Funnel plot for cancer metastasis.

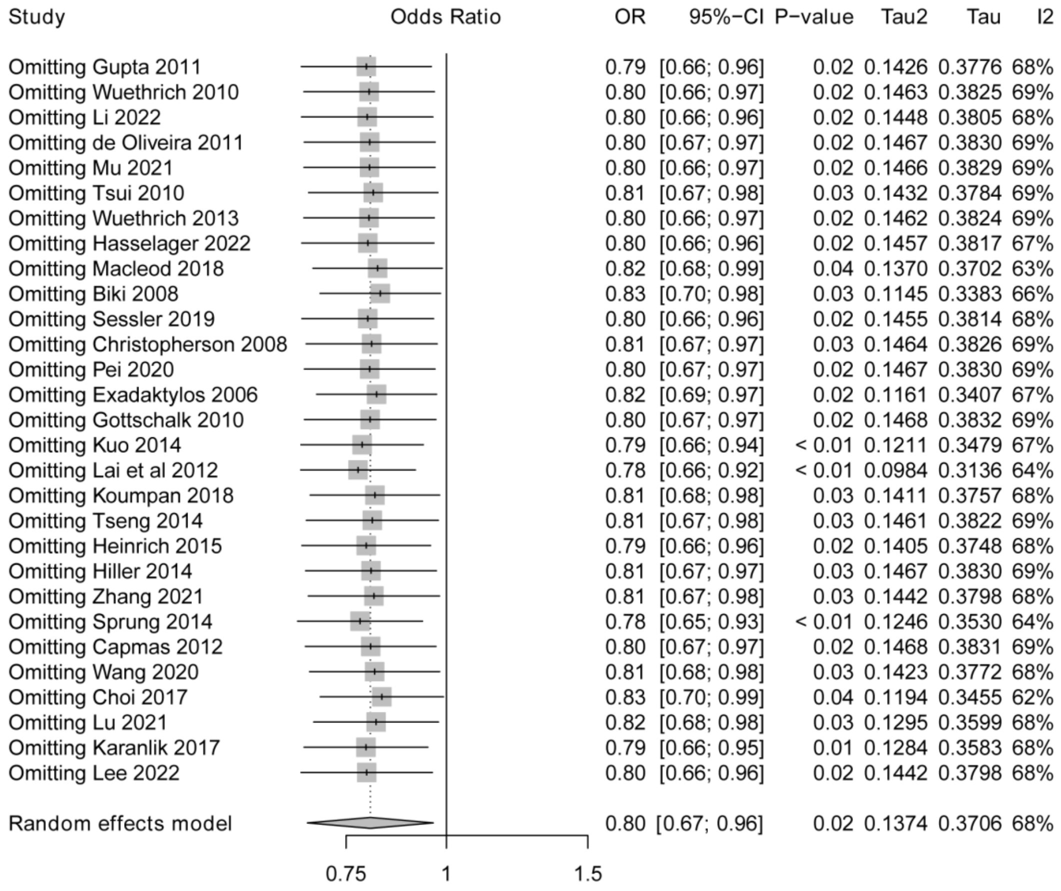

S Figure 3 recurrence-sensitivity

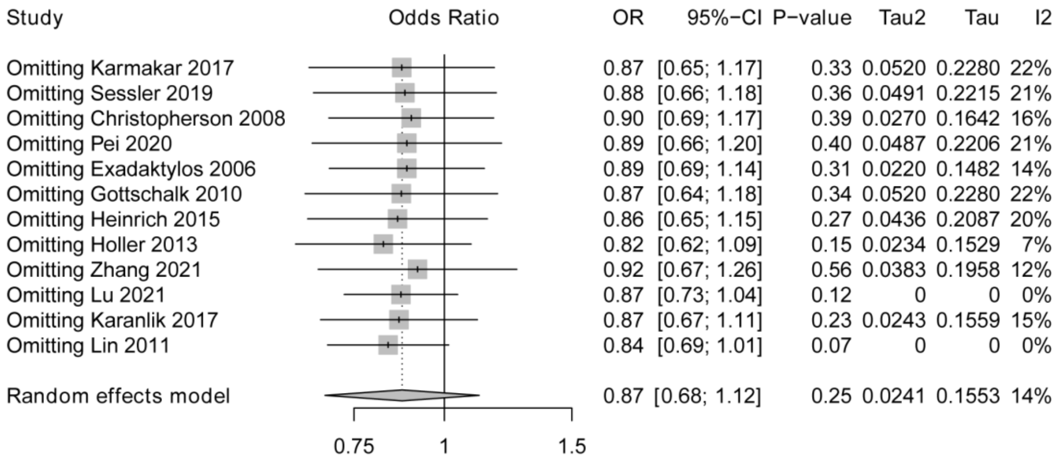

S Figure 5 metastasis-sensitivity

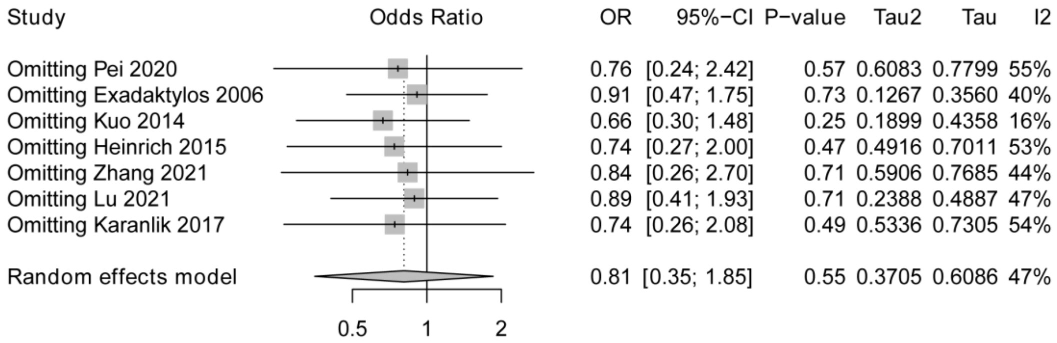

S Figure 6 local_recurrence-sensitivity

Title: Anesthesia type may impact on cancer recurrence and metastasis after cancer surgery: a meta-analysis

Date:2022-08-29

PubMed

		Search strategy	Numbers
**Patient**	**#1**	"Neoplasms"[Mesh]	3723902
	**#2**	((((((((((((Cancer[Title/Abstract]) OR (carcinoma[Title/Abstract])) OR (Tumor[Title/Abstract])) OR (Neoplasm[Title/Abstract])) OR (Tumors[Title/Abstract])) OR (Neoplasia[Title/Abstract])) OR (Neoplasias[Title/Abstract])) OR (Cancers[Title/Abstract])) OR (Malignant Neoplasm[Title/Abstract])) OR (Malignancy[Title/Abstract])) OR (Malignancies[Title/Abstract])) OR (Malignant Neoplasms[Title/Abstract])) OR (Neoplasm, Malignant[Title/Abstract])) OR (Neoplasms, Malignant[Title/Abstract])	3448685
	**#3**	**#1 OR #2**	4728061
**Intervention**	**#4**	"Anesthesia, Local"[Mesh]	18901
	**#5**	esia, Regional[Title/Abstract])) OR (Anesthesia,Local[Title/Abstract])) OR (Anesthesia, Infiltration[Title/Abstract])) OR (Infiltration Anesthesia[Title/Abstract])) OR (Neural Therapy of Huneke[Title/Abstract])) OR (Huneke Neural Therapy[Title/Abstract])	185590
	**#6**	**#4 OR #5**	194141
**Outcome**	**#7**	(((((Recurrence [Title/Abstract]) OR (Metastasis[Title/Abstract])) OR (Lymph node metastasis[Title/Abstract])) OR (Survival analysis[Title/Abstract])) OR (Recurrence Free Survival[Title/Abstract])) OR (prognosis[Title/Abstract])	1011091
**P+I+O**	**#8**	**#3 AND #6 AND #7**	2377

Cochrane Library

Cochrane		Search strategy	Numbers
**Patient**	**#1**	MeSH descriptor: [Neoplasms] explode all trees	89518
	**#2**	(neoplasms):ti,ab,kw OR(Cancer):ti,ab,kw OR(carcinoma):ti,ab,kw OR(Tumor):ti,ab,kw OR(Neoplasm):ti,ab,kw OR(Tumors):ti,ab,kw OR(Neoplasia):ti,ab,kw OR(Neoplasias):ti,ab,kw OR(Cancers):ti,ab,kw OR(Malignant Neoplasm):ti,ab,kw OR(Malignancy):ti,ab,kw OR(Malignancies):ti,ab,kw OR(Malignant Neoplasms):ti,ab,kw OR(Neoplasm, Malignant):ti,ab,kw OR(Neoplasms, Malignant):ti,ab,kw	242025
	**#3**	**#1** OR **#2**	252406
**Intervention**	**#4**	MeSH descriptor: [Anesthesia, Local] explode all trees	2285
	**#5**	(Regional Anesthesia):ti,ab,kw OR(Local Anesthesia):ti,ab,kw OR(Epidural Anesthesia):ti,ab,kw OR(neuraxial anesthesia):ti,ab,kw OR(spinal anesthesia):ti,ab,kw OR(Anesthetic Technique):ti,ab,kw OR(Anesthesia):ti,ab,kw OR(Anesthesia, Regional):ti,ab,kw OR(Anesthesia,Local):ti,ab,kw OR(Anesthesia, Infiltration):ti,ab,kw OR(Infiltration Anesthesia):ti,ab,kw OR(Neural Therapy of Huneke):ti,ab,kw OR(Huneke Neural Therapy):ti,ab,kw	77929
	**#6**	**#4 OR #5**	77929
**Outcome**	**#7**	(Recurrence):ti,ab,kw OR (Metastasis):ti,ab,kw OR (Lymph node metastasis):ti,ab,kw OR (Survival analysis):ti,ab,kw OR (Recurrence Free Survival):ti,ab,kw OR (prognosis):ti,ab,kw	138637
**P+I+O**	**#8**	**#3** AND **#7**	568

Embase

		Search strategy	Numbers
**Patient**	**#1**	'neoplasm'/exp	5718353
	**#2**	'neoplasms':ab,ti or 'Cancer':ab,ti or 'carcinoma':ab,ti or 'Tumor':ab,ti or 'Neoplasm':ab,ti or 'Tumors':ab,ti or 'Neoplasia':ab,ti or 'Neoplasias':ab,ti or 'Cancers':ab,ti or 'Malignant Neoplasm':ab,ti or 'Malignancy':ab,ti or 'Malignancies':ab,ti or 'Malignant Neoplasms':ab,ti or 'Neoplasm, Malignant':ab,ti or 'Neoplasms, Malignant':ab,ti	473108
	**#3**	**#1 OR #2**	6577582
**Intervention**	**#4**	'local anesthesia'/exp	46438
	**#5**	'Regional Anesthesia':ab,ti or 'Local Anesthesia':ab,ti or 'Epidural Anesthesia':ab,ti or 'neuraxial anesthesia':ab,ti or 'spinal anesthesia':ab,ti or 'Anesthetic Technique':ab,ti or 'Anesthesia':ab,ti or 'Anesthesia, Regional':ab,ti or 'Anesthesia,Local ':ab,ti or 'Anesthesia, Infiltration':ab,ti or 'Infiltration Anesthesia':ab,ti or 'Neural Therapy of Huneke':ab,ti or 'Huneke Neural Therapy':ab,ti	233960
	**#6**	**#4 OR #5**	260571
**Outcome**	**#7**	'Recurrence':ab,ti or 'Metastasis':ab,ti or 'Lymph node metastasis':ab,ti or 'Survival analysis':ab,ti or 'Recurrence Free Survival':ab,ti or 'prognosis':ab,ti	1452933
**P+I+O**	**#8**	**#3 AND #6 AND #7**	4425

Supplementary Table 2 Quality assessment of included studies by NOS

Study	1	2	3	4	5	6	7	8	Total
Lee 2022	1	1	1	1	2	1	0	0	7
Lin 2011	0	1	1	1	1	1	1	0	6
Karanlik 2017	1	0	0	1	1	1	1	0	5
Lu 2021	0	1	1	1	2	1	1	1	8
Choi 2017	1	1	1	1	1	1	1	0	7
Wang 2020	1	1	1	1	2	1	0	0	7
Capmas 2012	0	1	1	1	1	1	0	1	6
Sprung 2014	1	1	1	1	2	1	1	1	9
Zhang 2021	1	1	1	1	2	1	1	1	9
Holler 2013	1	1	1	1	0	1	1	0	7
Hiller 2014	0	1	1	1	2	1	0	1	7
Heinrich 2015	0	1	1	1	2	1	1	0	7
Tseng 2014	1	1	1	1	2	1	1	1	9
Koumpan 2017	0	1	1	1	0	1	1	0	5
Lai 2011	0	1	1	1	2	1	1	0	7
Kuo 2014	0	1	1	1	2	1	0	1	7
Gottschalk 2010	1	1	1	1	1	1	1	0	7
Exadaktylos 2006	0	1	1	1	2	1	0	1	7
Pei 2020	0	1	1	1	2	1	0	1	7
Biki 2008	0	1	1	1	1	1	1	0	6
Macleod 2018	1	1	1	1	1	1	1	0	7
Hasselager 2022	1	1	1	1	0	1	1	0	6
Wuethrich 2013	0	1	1	1	2	1	1	0	7
Mu 2021	0	1	1	1	2	1	0	1	7
de Oliveira 2011	0	1	1	1	2	1	1	0	7
Wuethrich 2010	1	1	1	1	1	1	1	0	7
Gupta 2011	1	1	1	1	1	1	0	0	6

Cohort study: 1. Representativeness of the exposed cohort; 2. Selection of the non-exposed cohort; 3. Ascertainment of exposure; 4. Demonstration that outcome of interest was not present at start of study. 5. Comparability of cohorts on the basis of the design analysis; 6. Assessment of outcomes; 7. Was follow-up long enough for
outcomes to occur; 8. Adequacy of follow-up of cohorts.

Case-control study: 1. Is the case definition adequate? 2. Representativeness of the cases; 3. Selection of Controls; 4. Definition of Controls; 5. Comparability of cases and controls on the basis of the design or analysis; 6. Ascertainment of exposure; 7. Same method of ascertainment for cases and controls; 8. non-Response rate.

Supplementary table3 Quality assessment of included studies by ROB 2.0

Study	Randomization process	Deviations from intended interventions	Missing outcomes data	Measurement of the outcome	Selection of the reported results	Overall bias
Li, 2022	Some concerns	Low	Low	Low	Low	Low
Tsui,2010	Low	Low	Some concerns	Low	Low	Low
Karmarkar,2017	Low	Low	Some concerns	Low	Low	Low
Seller,2019	Some concerns	Low	Some concerns	Low	Low	Low
Chistopherson,2008	Some concerns	High	High	Low	Low	High

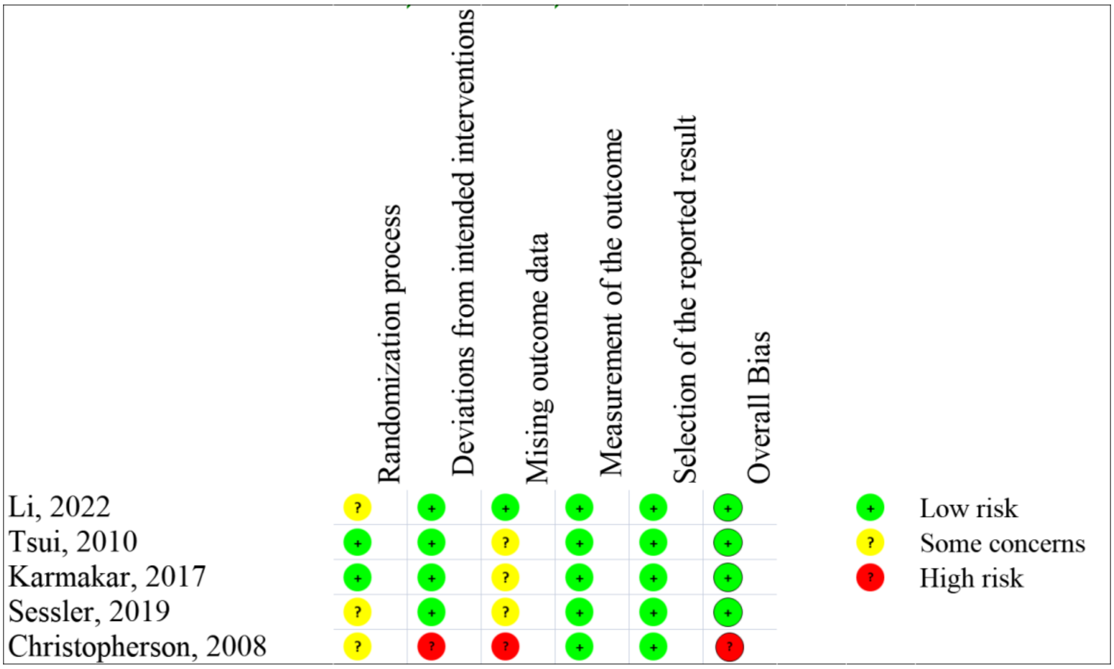
Randomization process
Deviations from intended interventions
Mising outcome data
Measurement of the outcome
Selection of the reported result
Overall Bias
Li, 2022
Tsui, 2010
Karmakar, 2017
Sessler, 2019
Christopherson, 2008
Low risk
Some concerns
High risk
